# Impact of Regional Mobility on Air Quality during COVID-19 Lockdown in Mississippi, USA Using Machine Learning

**DOI:** 10.3390/ijerph20116022

**Published:** 2023-05-31

**Authors:** Francis Tuluri, Reddy Remata, Wilbur L. Walters, Paul B. Tchounwou

**Affiliations:** 1 Department of Industrial Systems & Technology, Jackson State University, Jackson, MS 39217, USA; 2 Department of Atmospheric Sciences, Jackson State University, Jackson, MS 39217, USA; remata.s.reddy@jsums.edu; 3 College of Sciences, Engineering & Technology, Jackson State University, Jackson, MS 39217, USA; wilbur.l.walters@jsums.edu; 4 RCMI Center for Health Disparities Research, Jackson State University, Jackson, MS 39217, USA; 5 RCMI Center for Urban Health Disparities Research and Innovation, Morgan State University, Baltimore, MD 21251, USA

**Keywords:** COVID-19, air quality, transportation and mobility, python programming, statistical descriptive analysis, machine learning modeling

## Abstract

Social distancing measures and shelter-in-place orders to limit mobility and transportation were among the strategic measures taken to control the rapid spreading of COVID-19. In major metropolitan areas, there was an estimated decrease of 50 to 90 percent in transit use. The secondary effect of the COVID-19 lockdown was expected to improve air quality, leading to a decrease in respiratory diseases. The present study examines the impact of mobility on air quality during the COVID-19 lockdown in the state of Mississippi (MS), USA. The study region is selected because of its non-metropolitan and non-industrial settings. Concentrations of air pollutants—particulate matter 2.5 (PM2.5), particulate matter 10 (PM10), ozone (O_3_), nitrogen oxide (NO_2_), sulfur dioxide (SO_2_), and carbon monoxide (CO)—were collected from the Environmental Protection Agency, USA from 2011 to 2020. Because of limitations in the data availability, the air quality data of Jackson, MS were assumed to be representative of the entire region of the state. Weather data (temperature, humidity, pressure, precipitation, wind speed, and wind direction) were collected from the National Oceanic and Atmospheric Administration, USA. Traffic-related data (transit) were taken from Google for the year 2020. The statistical and machine learning tools of R Studio were used on the data to study the changes in air quality, if any, during the lockdown period. Weather-normalized machine learning modeling simulating business-as-scenario (BAU) predicted a significant difference in the means of the observed and predicted values for NO_2_, O_3_, and CO (*p* < 0.05). Due to the lockdown, the mean concentrations decreased for NO_2_ and CO by −4.1 ppb and −0.088 ppm, respectively, while it increased for O_3_ by 0.002 ppm. The observed and predicted air quality results agree with the observed decrease in transit by −50.5% as a percentage change of the baseline, and the observed decrease in the prevalence rate of asthma in MS during the lockdown. This study demonstrates the validity and use of simple, easy, and versatile analytical tools to assist policymakers with estimating changes in air quality in situations of a pandemic or natural hazards, and to take measures for mitigating if the deterioration of air quality is detected.

## 1. Introduction

Emissions released in the air by industries, motor vehicles, forest fires, power plants, and construction sites contain harmful pollutants. Among these pollutants, the primary air contaminants of health concern are particulate matter 2.5 microns in size (PM2.5), particulate matter 10 microns in size (PM10), nitrogen dioxide (NO_2_), ozone (O_3_), sulfur dioxide (SO_2_), and carbon monoxide (CO) [1,2]. PM2.5 can get into the lungs or can be absorbed into the blood and affect human health [3,4]. Air quality plays a dominant role in controlling respiratory diseases such as asthma, lung cancer, and chronic obstructive pulmonary disease (COPD). The World Health Organization (WHO) reports that ambient air pollution led to 4.2 million deaths worldwide in the year 2016 [5]. Of the total, 25% of deaths were caused by COPD, and 26% were caused by respiratory infection. Respiratory illness caused by coronavirus (COVID-19) can also be aggravated by air pollution [6,7].

The WHO declared COVID-19 as a pandemic on the 11th of March 2020 [8] due to the increasing COVID-19 incidence in different places worldwide [9]. Lockdown measures were taken by different countries initially during the period from March to June 2020 to mitigate the spread of COVID-19 incidence [10,11,12]. A recent survey of the literature shows an increase in the study of the effects of the COVID-19 pandemic lockdown measures on human living [13,14]. Because of its large-scale influence in every area of business and life, there has also been an increase in the study of the effect of the COVID-19 pandemic lockdown on air quality and health. A few examples of similar studies are worth noting.

A study of the effects of quarantine and social distancing in four megacities (Sao Paulo in Brazil, Paris in France, and Los Angeles and New York in the United States) on four air pollutants—CO, O_3_, PM2.5, and NO_2_—showed a decrease in their levels during March 2020 compared to their ambient concentrations in 2015–2019 [15]. Venter et al. [16] reported that during the lockdown, there was a reduction in the population-weighted concentration of nitrogen dioxide and particulate matter levels by about 60% and 31%, respectively, in 34 countries, with mixed effects on ozone. They pointed out that the reductions in the transportation sector emissions were largely responsible for the NO_2_ anomalies. In a comprehensive study on 87 capital, industrial, and polluted cities around the world, Sarmadi, M et al. [17] observed large changes in the air quality index (AQI) variation before and after the onset of the COVID-19 pandemic—the overall changes of AQI-PM2.5, AQI-PM10, and AQI-NO_2_ in 2020 were −7.36%, −17.52%, and −20.54% compared to 2019. Among 11 cities globally, Shi, V et al. [18] reported that PM2.5 did not show a decrease in many cities except in Wuhan, Rome, and Los Angeles, and exhibited a more complex response to the lockdown measures. They pointed out that the COVID-19 lockdown restrictions showed changes in the air pollutant levels, but the changes were smaller than expected. Jiang, Z et al. [19] showed that NO_2_ and PM2.5 decreased by 27 % and 15 %, respectively, in southern California in the 5 weeks after the stay-at-home orders. Campbell et al. [20] used ground, satellite-based observations of NO_2_, and National Air Quality Forecasting Capability (NAQFC) modeling to study the COVID-19 lockdown over the US. They showed that there were widespread decreases in NOx emissions among different states. Hammer et al. [21] mapped global PM2.5 concentrations from January to April 2020 using a combination of satellite data, simulation, and ground-based observations. They examined the PM2.5 concentrations during the lockdown periods in 2020 compared to the same periods from 2018 to 2019 and found a widespread decrease in PM2.5 over different regions. A study on Los Angeles air quality [22,23] reported a decrease in the concentrations of air pollutants during the COVID-19 lockdown. Yummin et al. [24] also demonstrated short-term health effects in China associated with the decrease in air pollutants during the COVID-19 lockdown.

The present study aims to examine the impact of mobility on air quality during the COVID-19 lockdown in MS. The study region was selected because of its non-metropolitan and non-industrial settings, unlike states such as Texas, New York, Ohio, Michigan, and California with heavy traffic and industries. Hence, it is interesting to study the impact of the COVID-19 lockdown on air quality in the region. The study hypothesizes that, in principle, the COVID-19 lockdown does affect the environment and air quality even in non-metropolitan and non-industrial environments. Statistical and machine learning tools were used to analyze the air quality data to capture changes in the air quality, if any, due to the COVID-19 lockdown. Machine learning modeling was used to predict air pollutant concentrations during the lockdown period, by subtracting the influence of weather changes that might mask the true emission changes [18,25,26,27,28,29,30,31,32].

## 2. Materials and Methods

Air quality data in the study region were obtained from the US Environmental Protection Agency (EPA) [33] for the period from 2011 to 2020 because of the availability of the data. The air quality data consist of the concentrations of six pollutants—PM2.5, PM10, O_3_, NO_2_, SO_2_, and CO. Because of the limited number of air quality collecting stations and large missing data, the air quality data are assumed to be representative of the entire region of the state.

Weather data were collected from the National Oceanic and Atmospheric Administration (NOAA) [34]. The weather data consist of the values of six meteorological variables—temperature, humidity, pressure, precipitation, wind speed, and wind direction.

Traffic is related to vehicle emissions such as nitrogen oxide (NO_X_), CO, and black carbon (BC) that affect air quality. Traffic-related data (transit) for the study region were collected from Google community mobility reports [35,36,37] and from ‘Our World Data’ [38]. The COVID-19 lockdown in MS, USA was implemented from 15 March to 1 June 2020 [39] in terms of transportation restrictions.

This study was carried out to examine whether the COVID-19 lockdown affected the environment and air quality even in a region of non-metropolitan and not industrial settings. Exploratory data analysis was carried out using statistical methods. Machine learning modeling using weather normalization simulating BAU was carried out to make predictions of air quality. The computations of statistical analysis and machine learning modeling were performed using R programming in R-Studio [40] running on a PC with an Intel CORE i7 processor, 16 GB RAM, and 500 GB hard drive memory.

## 3. Results

The results of the time series study are given in Section 3.1, and the results of the machine learning model are given in Section 3.2.

### 3.1. Time Series Study and Results

The observed data on air pollutants, weather, and mobility were studied and organized as follows:

3.1.1. Time series study of air pollutants
3.1.2. Monthly and day-of-the-week variations of air pollutants
3.1.3. Time series study of weather
3.1.4. Time series study of mobility in MS, 2020

#### 3.1.1. Time Series Study of Air Pollutants

**Figure 1:**
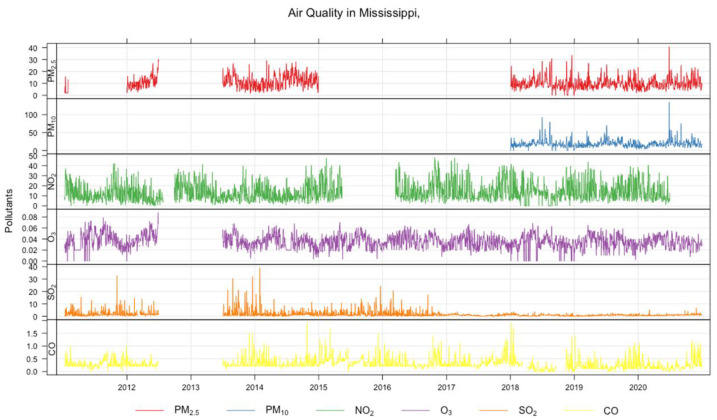
Time series of air pollutants arranged in subplots consisting of six rows. From the top, row 1, row 2, row 3, row 4, row 5, and row 6 represent the time variation of PM2.5, PM10, NO_2_, O_3,_ SO_2_, and CO, respectively. The units of measurement for the air pollutants are µg/m^3^, µg/m^3^, ppm, ppb, ppb, and ppm for PM2.5, PM10, O_3_, NO_2_, SO_2_, and CO, respectively.

The time series of air pollutants are shown as a grid plot arranged in subplots of six rows in Figure 1. From the top, row 1, row 2, row 3, row 4, row 5, and row 6 show the time variations of PM2.5, PM10, NO_2_, O_3_, SO_2_, and CO, respectively. The units of measurement for the air pollutants are µg/m^3^, µg/m^3^, ppm, ppb, ppb, and ppm for PM2.5, PM10, O_3_, NO_2_, SO_2_, and CO, respectively.

The results of the descriptive statistics on the air pollutant data are shown in Table 1. The quantities describing the distribution of data are minimum (Min), maximum (Max), median, mean, first quartile (25%), third quartile (75%), and missing data (NAN). The first three quartiles determine the interquartile range, which is a measure of the variability of data around the median. The mean values of PM2.5, PM10, O_3_, NO_2_, SO_2_, and CO are 10.36 µg/m^3^, 18.7 µg/m^3^, 0.0355 ppm, 13.69 ppm, 1.59 ppb, and 0.31ppm, respectively. The maximum values of these pollutants are 40.70 µg/m^3^, 135.0 µg/m^3^, 0.0880 ppm, 48.30 ppb, 39.10 ppb, and 1.90 ppm, respectively.

During the study period, the correlations between the air pollutants are presented in Table 2. The highest correlation is 0.82 between PM2.5 and PM10. The correlation between NO_2_ and PM2.5 is 0.20. Overall, a positive correlation is observed between the air pollutants, though small.

#### 3.1.2. Monthly and Day-of-the-Week Averages of Air Quality

**Figure 2:**
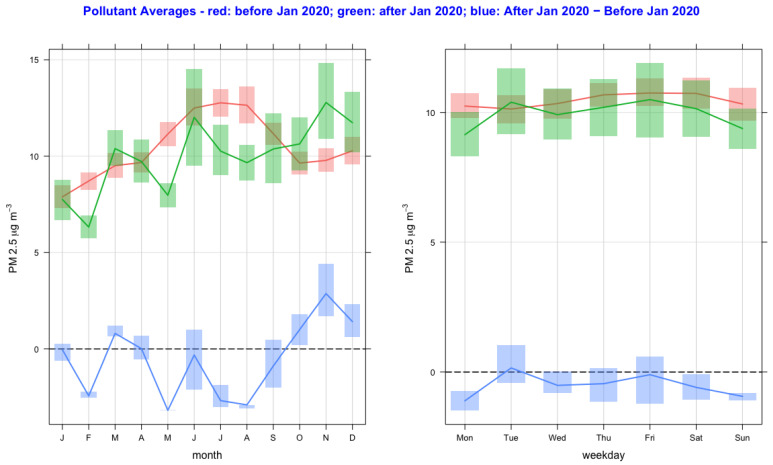
Time variation of PM2.5 concentrations before and after the lockdown year (2020), and their difference as a function of monthly and day-of-the-week averages. The colors green, red, and blue represent the periods during the lockdown year, before the lockdown year, and their difference, respectively. The full lines and vertical bars represent mean and 95% confidence intervals, respectively.

**Figure 3:**
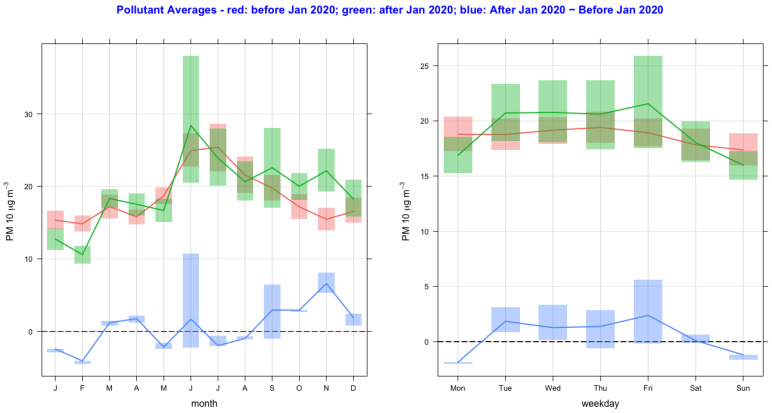
Time variation of PM10 concentrations before and after the lockdown year (2020), and their difference as a function of monthly and day-of-the-week averages. The colors green, red, and blue represent the periods during the lockdown year, before the lockdown year, and their difference, respectively. The full lines and vertical bars represent mean and 95% confidence intervals, respectively.

**Figure 4:**
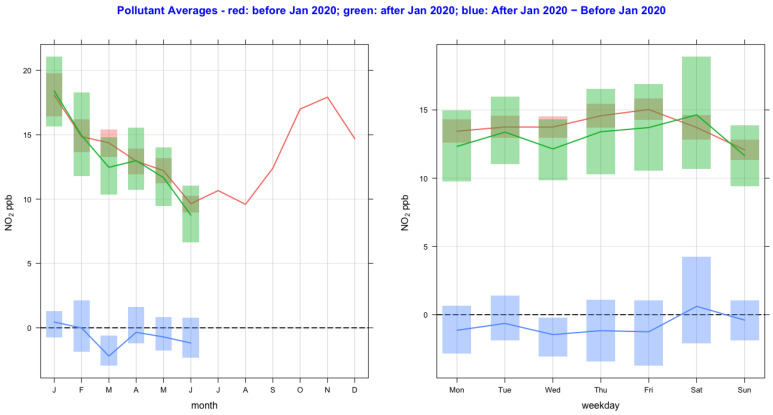
Time variation of NO_2_ concentrations before and after the lockdown year (2020), and their difference as a function of monthly and day-of-the-week averages. The colors green, red, and blue represent the periods during the lockdown year, before the lockdown year, and their difference.

**Figure 5:**
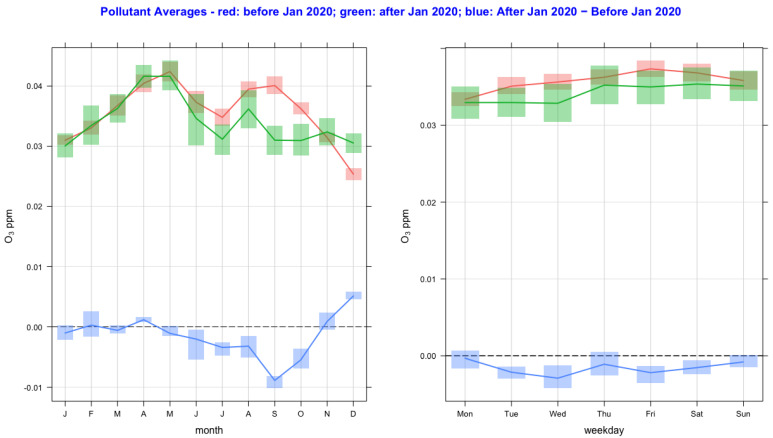
Time variation of O_3_ concentrations before and after the lockdown year (2020), and their difference as a function of monthly and day-of-the-week averages. The colors green, red, and blue represent the periods during the lockdown year, before the lockdown year, and their difference.

**Figure 6:**
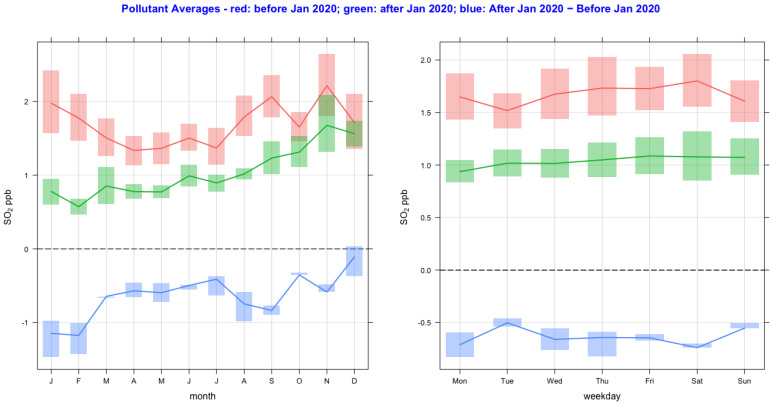
Time variation of SO_2_ concentrations before and after the lockdown year (2020), and their difference as a function of monthly and day-of-the-week averages. The colors green, red, and blue represent the periods during the lockdown year, before the lockdown year, and their difference.

**Figure 7:**
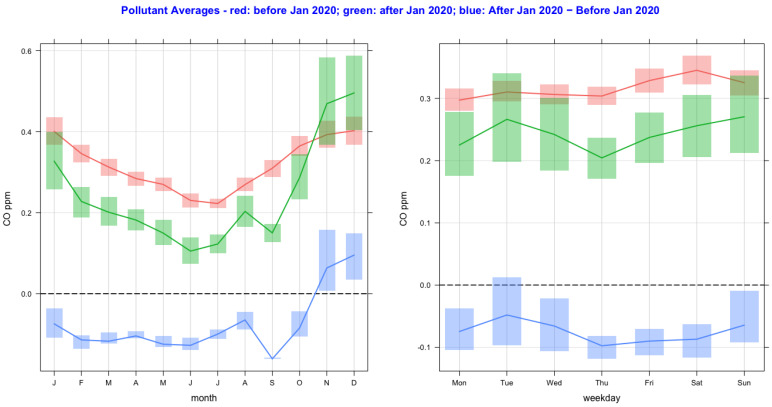
Time variation of CO concentrations before and after the lockdown year (2020), and their difference as a function of monthly and day-of-the-week averages. The colors green, red, and blue represent the periods during the lockdown year, before the lockdown year, and their difference.

The time variation of air pollutant concentrations before and after the lockdown year (2020) and their difference as a function of monthly and day-of-the-week averages are shown in Figure 2, Figure 3, Figure 4, Figure 5, Figure 6 and Figure 7 for the air pollutants PM2.5, PM10, NO_2_, O_3,_ SO_2_, and CO, respectively. For each figure, column 1 and column 2 show monthly and day-of-the-week averages, respectively. The time duration considered for the lockdown year is from 1 January to 31 December 2020. For the pre-lockdown period, the time duration considered is from 1 January 2011 to 31 December 2019. The colors green, red, and blue represent the periods during the lockdown year, before the lockdown year, and their difference, respectively. During the lockdown period in 2020 (March to June), the difference line shows a decrease in the pollutant concentrations except for PM10 and O_3_. PM2.5 shows a decrease in some months and an increase in other months, and much the same occurs with the rest of the pollutants, except for SO_2_, which shows a year-round value below zero. In Figure 2 and Figure 3, it is also observed that in the midweek (Wednesday and Thursday), the difference line shows a decrease in the pollutant concentrations except for PM10. Perhaps the lockdown largely restricted mobility during the week, compared to the weekend, when people might have more mobility to procure weekly provisions.

#### 3.1.3. Time Series Study of Meteorological Trends

**Figure 8:**
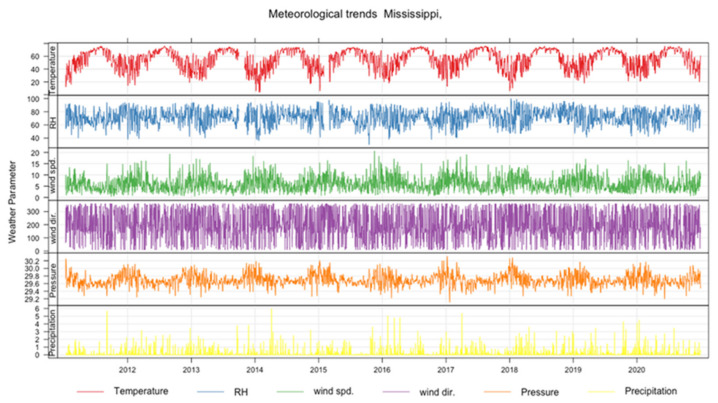
The time series of meteorological parameters in a grid plot arranged as subplots of six rows. From the top, row 1, row 2, row 3, row 4, row 5, and row 6 show the time variation of temperature, humidity, wind speed, wind direction, pressure, and precipitation, respectively. The units for the variables are Fahrenheit, percentage, mph, degrees, in. Hg, and inches, respectively.

The time series of the meteorological parameters are shown as a grid plot arranged in subplots of six rows in Figure 8. From the top, row 1, row 2, row 3, row 4, row 5, and row 6 show the time variation of temperature, humidity, wind speed, wind direction, pressure, and precipitation, respectively. The units for the variables are Fahrenheit, percentage, mph, degrees, in. Hg, and inches, respectively. The time series of the temperature, humidity, pressure, and wind speed show general seasonality patterns. For example, the temperatures are low in the winter and high in the summer. The relative humidity pattern is generally low when the temperatures are high, showing a mirror image. The pressure and wind speed show a general pattern of highs in the winter and lows in the summer. The precipitation shows variability, but a high amount of rainfall is normally associated with cool seasons. However, the impact of the weather on the air quality during lockdown can only be established by eliminating seasonal contributions using machine learning.

The results of the descriptive statistics on the meteorological data are shown in Table 3. The minimum and maximum values of the temperature, humidity, pressure, wind speed, wind direction, and precipitation are 4.00 °F, 30.00%, 29.11 in Hg, 0.11 mph, 10.0°, and 0.00 in; and 76.0 °F, 100.0%, 30.32 in Hg, 20.6 mph, 360°, and 5.97 in, respectively, with their mean being 55.44 °F, 71.55%, 29.70 in Hg, 6.07 mph, 196.3°, and 0.17 in, respectively.

During the study period, the correlations between the meteorological variables are presented in Table 4. In particular, the temperature has a positive correlation of 0.44 and 0.13 with humidity and precipitation, respectively, but shows a negative correlation of −0.63, −0.10, and −0.08 with pressure, wind speed, and wind direction, respectively.

#### 3.1.4. Mobility: Time Series Study and Results

**Figure 9:**
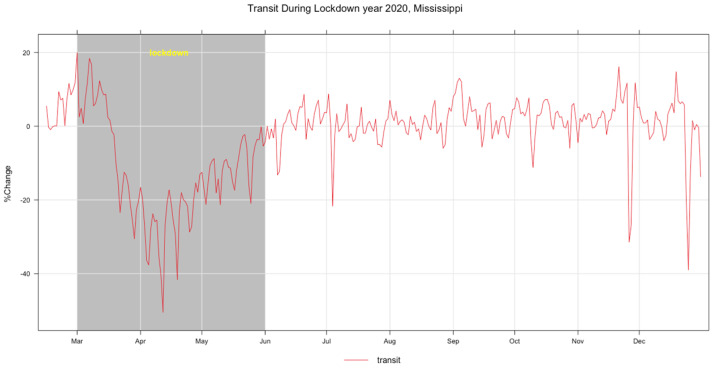
Time series plot of percentage change in transit, 2020.

Vehicle emissions are sources of air pollutants such as oxides of nitrogen (NO_X_), CO, and black carbon (BC) that affect air quality. Hence, transit (traffic-related) data were collected from the Google community mobility report [35,36,37]. Figure 9 shows the time series of the transit data as a percentage change based on a baseline [18,37], and its summary statistics are given in Table 5. The percentage changes in the mean, maximum, and minimum values of transit are −2.79, 20.00, and −50.50, respectively. The observed decrease in transit of −50.5% is expected to have contributed to the changes in the air quality during the COVID-19 lockdown.

### 3.2. Machine Learning Modeling: Business as Usual Scenario Model

Apart from the lockdown intervention on air quality, meteorological conditions also play a role in affecting air pollutant concentrations. Favorable weather conditions such as increased wind and rain lower the air pollutant concentrations more than on a normal day of the week, whereas unfavorable conditions of low winds and a stable atmosphere may elevate the concentrations. Quantifying the changes caused by the lockdown by comparing the air pollutant concentrations before and after the intervention (such as in Table 3) may lead to wrong conclusions, since the meteorological changes may mask the variation in concentrations caused by the intervention. Hence, it would be difficult to determine from the observed data whether the changes in the concentrations (increase or decrease) are caused by weather conditions or by the traffic regulations implemented during the lockdown. By using machine learning models, one can subtract the weather component from the observation to obtain weather-normalized data that show the underlying causes of the change in the concentrations simulating a business-as-usual scenario (BAU) [18,25,26,27,28,29,30,31,32]. Weather normalization can be achieved by using random forest (RF) regression models [41] via the ‘randomForest’ package in R [42]. “RF” regression is a type of ensemble learning method using many of what are known as “weak” predictors for building a forest of decision trees to obtain a good prediction accuracy [32].

In the present study, the weather normalization of Grange et al. was implemented using an RF model-based R programming package—rmweather [25,26,27]. The major instructions used to execute the model are as follows:

1. Install and run the packages in R studio [40].
2. Load and run the data set for each pollutant and the independent variables.
  - Features or independent variables.
    - Meteorological variables—temperature, humidity, pressure, wind speed, wind direction, and precipitation.
    - Temporal variables—year, month, day, weekday, season.
  - Target variable—pollutant concentration.
3. Run the random forest model for training and create a meteorological normalized trend.
  - Define the training data set period.
  - Split the data and the training set; the test is for validation.
    - Input features—meteorological and temporal variables.
  - Run the RF model using weather normalization.
    ii. For each day, resample the meteorological explanatory variables by repeating a certain number of times (say 300).
    iii. Aggregate the predicted values from each iteration to obtain the meteorological normalized concentration.
    iv. The estimated values represent the emission changes rather than the changes due to meteorological effects.
    v. Repeat resampling for every day in the data set.
  - Set the hyper-parameters of the model.
4. Check the model performance on the test part of the training set.
5. Plot variable importance and decide which variables are more important, and remove insignificant variables if required.
6. Run the model prediction and check if the model has suffered from overfitting.
7. Check for partial dependencies and remove missing variables.
8. Run the weather-normalized and trained RF model on the test data set (lockdown period) to obtain the predicted values of the pollutant concentrations.
9. Collect the observed and predicted pollutant values; compute the pollutant change due to the lockdown, and plot for visualization.

For each pollutant, the model was trained on the past data of meteorological and temporal variables for the period from 1 January 2011 to 29 February 2020. Beyond the training period (29 February 2020), the trained model was used to predict the pollutant concentration using observed meteorological variables to generate a “counterfactual” time series that represents the estimation of concentrations under a business-as-usual scenario.

**Figure 10:**
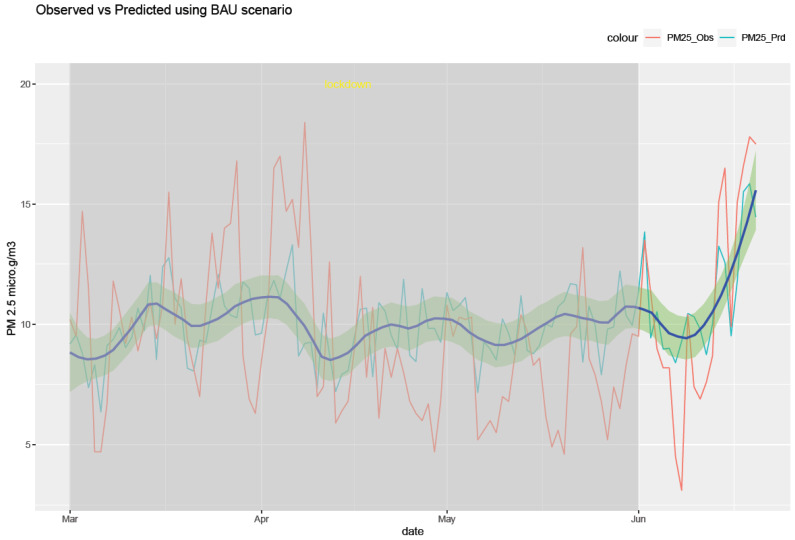
Weather normalized predictions of the pollutant PM 2.5. The blue line represents the smoothened plot of predicted values with the confidence intervals shown by the green shade.

**Figure 11:**
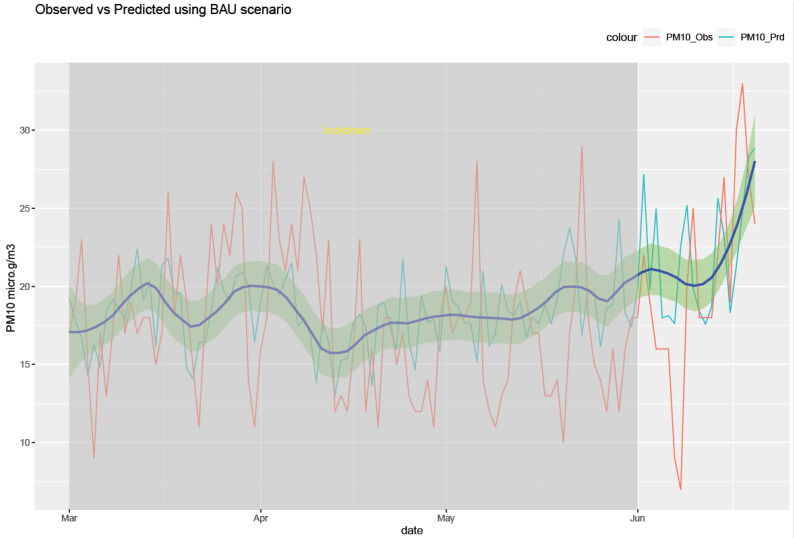
Weather normalized predictions of the pollutant PM 10. The blue line represents the smoothened plot of predicted values with the confidence intervals shown by the green shade.

**Figure 12:**
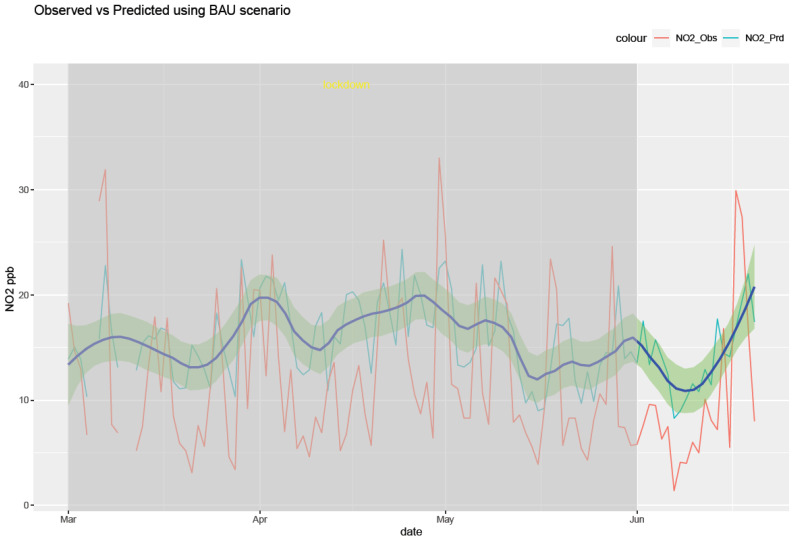
Weather normalized predictions of the pollutant NO_2_. The blue line represents the smoothened plot of predicted values with the confidence intervals shown by the green shade.

**Figure 13:**
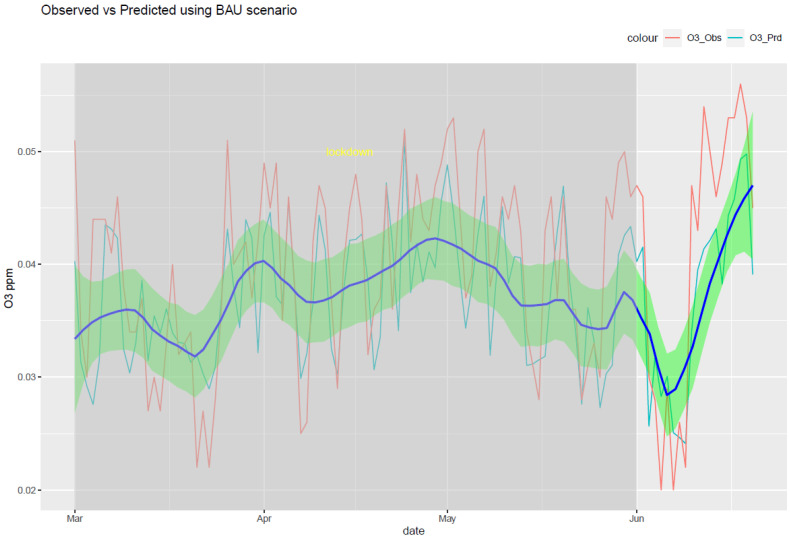
Weather normalized predictions of the pollutant O_3_. The blue line represents the smoothened plot of predicted values with the confidence intervals shown by the green shade.

**Figure 14:**
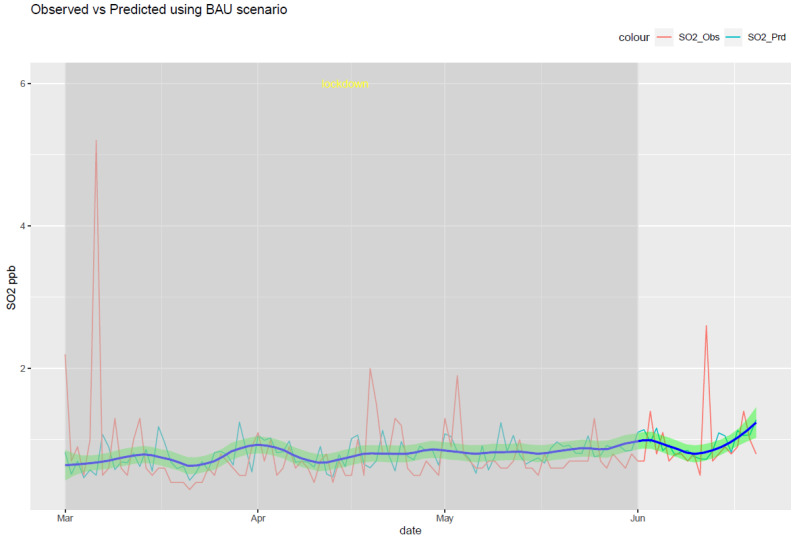
Weather normalized predictions of the pollutant SO_2_. The blue line represents the smoothened plot of predicted values with the confidence intervals shown by the green shade.

**Figure 15:**
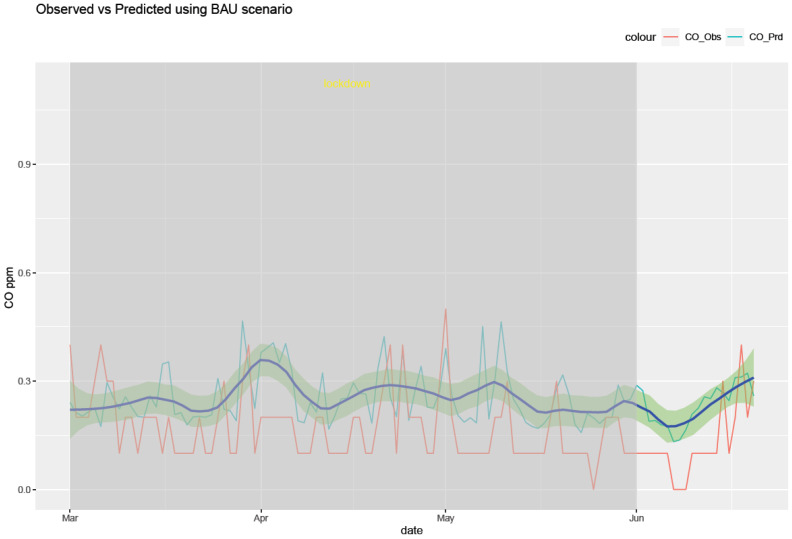
Weather normalized predictions of the pollutant CO. The blue line represents the smoothened plot of predicted values with the confidence intervals shown by the green shade.

**Figure 16:**
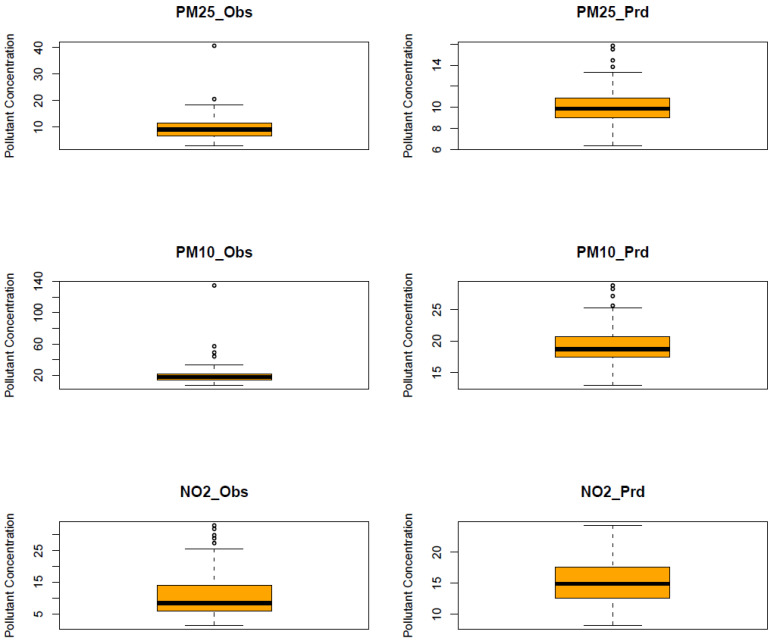
Boxplot distribution of observed and predicted air pollutants during a lockdown as subplots of three rows and two columns in pairs of observed and predicted concentrations for each pollutant. Starting from the top, the observed and predicted results of PM2.5, PM10, and NO_2_ are represented, respectively, by (row 1, column 1, and column 2), (row 2, column 1, and column 2), (row 3, column 1, and column 2). The underscore _obs and _prd represent the observed and predicted values, respectively, of each of the pollutants. The symbols PM25, PM10, and NO_2_, stand for the pollutants PM2.5, PM10, and NO_2_, respectively. The units of PM2.5, PM10, and NO_2_ are µg/m^3^, µg/m^3^, and ppb, respectively.

**Figure 17:**
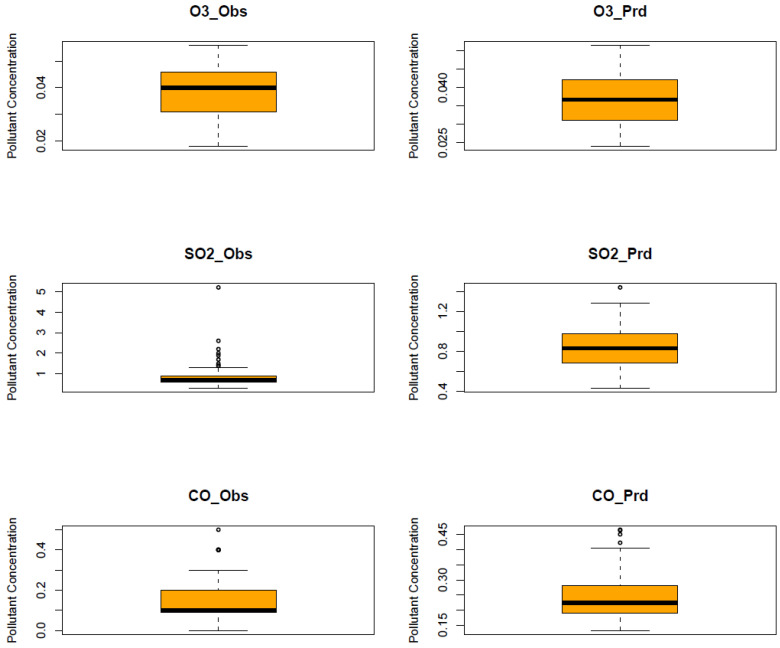
Boxplot distribution of observed and predicted air pollutants during a lockdown as subplots of three rows and two columns in pairs of observed and predicted concentrations for each pollutant. Starting from the top, the observed and predicted results of O_3_, SO_2_, and CO are represented, respectively, by (row 1, column 1, and column 2), (row 2, column 1, and column 2), (row 3, column 1, and column 2). The underscore _obs and _prd represent the observed and predicted values, respectively, of each of the pollutants. The symbols O_3_, SO_2_, and CO, stand for the pollutants O_3_, SO_2_, and CO, respectively. The units of O_3_, SO_2_, and CO, are ppm, ppb, and ppm, respectively.

The RF model was run for each of the six air pollutants (PM2.5, PM10, O_3_, NO_2_, SO_2_, and CO). The meteorological features of the model are temperature, humidity, pressure, precipitation, wind speed, and wind direction. The temporal features consist of a trend term (Unix date), a seasonal term (Julian day), a weekday, a week, and a month. The results of the model are shown in Figure 10, Figure 11, Figure 12, Figure 13, Figure 14 and Figure 15 for the observed and predicted results of PM2.5, O_3_, PM10, SO_2_, NO_2_, and CO, respectively. In the figures, the blue line represents the smoothened plot of predicted values with the confidence intervals shown by the green shade. The boxplot distribution of the observed and predicted concentrations for each air pollutant is shown in Figure 16 and Figure 17 as subplots of three rows and two columns in pairs of the observed and predicted concentrations for each pollutant. In Figure 16, starting from the top, the observed and predicted results of PM2.5, PM10, and NO_2_ are represented, respectively, by (row 1, column 1, and column 2), (row 2, column 1, and column 2), (row 3, column 1, and column 2). In Figure 17, starting from the top, the observed and predicted results of O_3_, SO_2_, and CO are represented, respectively, by (row 1, column 1, and column 2), (row 2, column 1, and column 2), (row 3, column 1, and column 2). The boxplot distribution summary statistics are shown in Table 6, where the underscore _obs and _prd represent the observed and predicted values, respectively, of each of the pollutants. The RF hyper-parameters used were kept the same for each model and were based on the best results—the number of trees used is 300, the number of predictors randomly sampled to determine each split (mtry) is two, and the minimum node size is five (see Table 7). The model metrics are also shown in Table 7. The root mean square error (RMSE) ranged between 7.56 and 0.008.

Statistical testing (*t*-test) was performed on the time series of the observed and predicted values for each pollutant to check if they were significantly distinct. The results of the statistical testing are shown in Table 8. For the case of NO_2_, O_3_, and CO, it was found that the means of the observed and predicted data are significantly distinct (*p* < 0.05). Due to the lockdown, the mean concentrations decreased for NO_2_ and CO by −4.1 ppb and −0.088 ppm, respectively, while the mean concentrations of O_3_ increased by 0.002 ppm (see Figure 16 and Figure 17.

For all the six air pollutants, the RF hyper-parameters used were kept the same—the number of trees used is 300, the number of predictors randomly sampled to determine each split (mtry) is two, and the minimum node size is five.

The units of PM2.5, PM10, O_3_, NO_2_, SO_2_, and CO are µg/m^3^, µg/m^3^, ppm, ppb, ppb, and ppm, respectively. ‘ci’ stands for confidence interval.

## 4. Discussion

Onyeaka et al. [13] reported that there was a reduction of up to 30% in environmental pollution as a result of half of the world’s population experiencing some form of lockdown, with an attendant reduction in mobility of up to 90%. A study of air quality during COVID-19 showed that the concentrations of PM 2.5 and PM10 decreased by 12% and 37% in Los Angeles, and 24% in New York, while NO_2_ decreased by 25% in Sao Paulo, 38% in Los Angeles, and 24% in New York [15]. In a comparative study of the impact of COVID-19 onset on air quality in several cities of the world, Washington, DC is reported to have a reduction in PM 2.5 by about 10% [17]. Mohammed et al. [43] reported that NO_2_ emissions were reduced by up to 30% based on the NASA satellite image over the northeastern USA before and after the lockdown. Chen et al. [44] completed a study of quarantine in China and found that PM2.5 dropped by 1.4 µg/m^3^ in Wuhan but decreased by 18.9 µg/m^3^across 367 cities, and NO_2_ dropped by 22.8 µg/m^3^ and 12.9 µg/m^3^ in Wuhan and China, respectively. Ghahremanloo et al. [45] reported a decrease in the percentage of PM 2.5 by 18%, 13.53%, and 20.7% in Boston, Detroit, and New York, respectively. In a study of air quality in urban sites in Spain, a slight reduction in PM10 (−4.1%) and PM2.5 levels (−2.3%) was observed during the lockdown, and a maximum reduction of above −50% was observed for NOx, whereas a maximum increase of 23.9% was observed for O_3_ in contrast with a decrease in NOx [46].

In the present study, weather-normalized RF machine modeling is used to take into account the influence of meteorological changes on the model. Lower temperatures (less than 45 °F) were observed with more frequent southerly winds than for other wind directions, while higher temperatures (greater than 70 °F) were associated more with northern and NW directions. Similar observations were also seen in the case of humidity. During the lockdown, there were southerly and SW (southwest) winds that were stronger and had greater frequency (number of occurrences) than in the pre-lockdown years. This would have an effect on the air pollutions by dispersion frequency, or the number of times the wind blew in a given direction. The weather-normalized RF model estimates the pollutant concentrations for the lockdown period by subtracting the weather influences and predicting the pollutant concentrations under the BAU scenario.

Using weather-normalized RF machine modeling, it was observed that the means of the observed and predicted air pollutant values are significantly distinct (*p* < 0.05) for the case of NO_2_, O_3_, and CO. Due to the lockdown, the mean concentrations decreased for NO_2_ (Figure 12, row 3 of Figure 16) and CO (Figure 15, row 3 of Figure 17) by −4.1 ppb and −0.088 ppm, respectively, while it increased for O_3_ (Figure 13, row of Figure 17) by 0.002 ppm, leading to a partial improvement in the air quality due to the lockdown. The decrease in the concentrations of NO_2_ reflects the restricted transit during lockdown because NO_2_ and diesel soot are directly related to automobile vehicular traffic. Since the RF model predictions are based on weather normalization, the decrease in NO_2_ and CO can be attributed to the lower emissions caused by less traffic during the lockdown. There were changes in the mean concentrations of particulate matter, but they cannot be accounted for since their *p*-values were found to be greater than 0.05. In the present study, there was an increase in the mean concentration of O_3_ by 0.002 ppm, similar to that observed by others [28,46]. Generally, ozone concentrations depend on its precursors such as nitrogen oxide (NOx). Fewer NOx emissions may increase O_3_ because it was broken down less frequently, but it is difficult to establish the lockdown impact on O_3_ based on NOx emissions only. Normally, asthma prevalence is associated with particulate matter. In MS, the percentage of adults reported as having asthma was 9.9% in 2019 but decreased to 8.9% in 2020 [47] which agrees with the model prediction of a decrease in the mean concentrations of PM2.5 (−0.1 µg/m^3^).

The methodology used in the present study is one of the two recent approaches developed during the post-pandemic period [28]. The two approaches differ in the way the effects of meteorology are analyzed to deduce pollutant changes during external influences such as the lockdown. In the first approach, a base case is used with a reference measurement period of the past, such as a similar period before the lockdown. Then, the changes in pollutant concentrations are deduced from the difference between base cases and the lockdown period. The method, although simple, does not completely eliminate the meteorological effects. The second approach uses predictive machine learning models to isolate lockdown intervention on the air pollutant concentration [25,26,27,28,29,30,31]. In the present study, the second approach is applied by comparing the predicted results of 2020 with actual observations made during the lockdown period. Hence, the results of the method used are a true measure of the relative changes for the lockdown intervention compared to the results obtained using the base case method. However, there are some other statistical, artificial neural network models and classification regression machine models that can be used to analyze the meteorological effects on air quality but were not explored presently and would be attempted later if adequate data size is available.

The present study confirms the hypothesis that, in principle, the COVID-19 lockdown affects the air quality even in non-metropolitan and non-industrial environments of MS. To study the spatial distribution of air quality, it would be good to have air quality data from multiple stations covering rural, urban, and traffic locations. Further, it is also required to have non-missing hourly observations for each station for validating the machine learning modeling accurately. In spite of these two limitations, the present study demonstrated an easy and simple methodology of using versatile tools of statistical and machine learning modeling for investigating the changes in air quality caused by pandemics or natural hazards. The methodology and results show the importance of taking mitigating measures for sustainable improvement in air quality.

## 5. Conclusions

Statistical and weather-normalized random forest modeling methods were used to study the impact of the COVID-19 lockdown on air quality in MS. Weather-normalized modeling mimics a business-as-usual scenario to establish the changes in the air quality as caused by the traffic regulations during the lockdown, by subtracting the weather component from the values of the observed air pollutants. For the pollutants NO_2_, CO, and O_3_, significant changes were found in their observed and predicted mean concentration values. The mean concentrations decreased for NO_2_ and CO but increased for O_3_. The decrease in the NO_2_ and CO concentrations reflects a partial improvement in the air quality due to the lockdown. The observed decrease in the pollutant concentration and the predicted air quality results show that the decrease in transit is associated with the decrease in asthma prevalence due to the lockdown. The decrease in NO_2_ and increase in O_3_ suggest different measures to mitigate these pollutants because one is a consequence of the other. The study examines the effect of lockdowns to control the COVID-19 pandemic on air quality, particularly in urban regions of non-metropolitan and non-industrial settings. The study demonstrates the utility of simple, easy, and versatile analytical tools to generate scientific data to assist policymakers in making informed decisions regarding the assessment and management of air quality during a pandemic or natural disaster.

## Figures and Tables

**Table 1:** Summary statistics of air quality.

Item	PM2.5 (µg/m^3^)	PM10 (µg/m^3^)	O_2_ (ppm)	NO_2_ (ppb)	SO_2_ (ppb)	CO (ppm)
Min.	0.00	0.00	0.00	0.00	00.0	0.00
1st Quartile	7.00	13.0	0.0200	7.00	0.50	0.20
Median	9.60	17.0	0.0350	11.20	0.90	0.30
Mean	10.36	18.7	0.0355	13.69	1.59	0.31
3rd Quartile	12.80	22.0	0.0440	18.60	1.70	0.40
Max.	40.70	135.0	0.0880	48.30	39.10	1.90
NA *N*	1374	2240	375	562	383	459

**Table 2:** Correlation between the air pollutants.

Item	PM2.5 (µg/m^3^)	PM10 (µg/m^3^)	O_3_ (ppm)	NO_2_ (ppb)	SO_2_ (ppb)	CO (ppm)
PM2.5	1.00	0.82	0.20	0.20	0.21	0.34
PM10	0.82	1.00	0.15	0.14	0.14	0.12
O_3_	0.20	0.15	1.00	0.19	0.11	0.10
NO_2_	0.20	0.14	0.19	1.00	0.10	0.55
SO_2_	0.21	0.14	0.11	0.10	1.00	0.29
CO	0.34	0.12	0.10	0.55	0.29	1.00

**Table 3:** Summary Statistics of Meteorological Variables.

Item	Temperature (°F)	Humidity (%)	Pressure (in. Hg)	Wind Speed (mph)	Wind Direction (Degrees)	Precipitation (in)
Min.	4.00	30.00	29.11	0.11	10.0	0.00
1st Quartile	45.00	64.00	29.60	3.70	130.0	0.00
Median	59.00	72.00	29.68	5.70	180.0	0.00
Mean	55.44	71.55	29.70	6.07	196.3	0.17
3rd Quartile	69.00	80.00	−29.78	8.00	300.0	0.04
Max.	76.00	100.00	30.32	20.6	360.0	5.97
NA *N*	82	63	2		40	

**Table 4:** Correlation Table: Meteorological Variables.

Item	Temperature (°F)	Humidity (%)	Pressure (in. Hg)	Wind Speed (mph)	Wind Direction (Degrees)	Precipitation (in)
Temperature	1.00	0.44	−0.63	−0.10	−0.08	0.13
Humidity	0.44	1.00	−0.43	0.08	−0.01	0.44
Pressure	−0.63	−0.43	1.00	−0.10	−0.09	−0.27
Wind Speed	−0.10	0.08	−0.10	1.00	0.05	0.24
Wind Direction	−0.08	−0.01	−0.09	0.05	1.00	0.00
Precipitation	0.13	0.44	−0.27	0.24	0.00	1.00

**Table 5:** Summary statistics of transit as percentage change by a baseline. Transit data were collected from the Google community mobility report [35,36,37] for the year 2020.

Item	Transit
Min.	−50.50
1st Quartile	−5.30
Median	0.36
Mean	−2.79
3rd Quartile	4.00
Max.	20.00

**Table 6:** Summary statistics of observed and model-predicted values of the air pollutants. The underscored _obs and _prd represent the observed and predicted values, respectively, of each of the pollutants. The symbols PM2.5, PM10, O_3_, NO_2_, SO_2_, and CO stand for the pollutants PM2.5, PM10, O_3_, NO_2_, SO_2_, and CO, respectively.

Item	PM25_Obs (µg/m^3^)	PM25_Prd (µg/m^3^)	PM10_Obs (µg/m^3^)	PM10_Prd (µg/m^3^)	O_3__Obs (ppm)	O_3__Prd (ppm)	NO_2__Obs (ppb)	NO_2__Prd (ppb)	SO_2__Obs (ppb)	SO_2__Prd (ppb)	CO_Obs (ppm)	CO_Prd (ppm)
Min.	3.10	6.35	7.00	12.98	0.018	0.024	1.40	8.27	0.300	0.430	0.000	0.132
1st Quartile	6,82	8.98	14.00	17.44	0.031	0.031	5.95	12.59	0.600	0.688	0.100	0.190
Median	9.10	9.85	18.00	18.73	0.040	0.036	8.40	14.92	0.700	0.830	0.100	0.223
Mean	9.97	10.07	19.69	19.17	0.038	0.036	11.23	15.33	0.837	0.835	0.156	0.244
3rd Quartile	11.50	10.87	22.0	20.60	0.046	0.042	14.00	17.60	0.900	0.971	0.200	0.278
Max.	40.70	15.85	135.0	28.85	0.056	0.051	33.00	24.33	5.200	1.439	0.500	0.466

**Table 7:** rmweather model statistics and metrics.

Item	PM2.5	PM10	NO_2_	O_3_	SO_2_	CO
R squared	0.332	0.298	0.310	0.489	0.188	0.488
RMSE	3.01	7.55	7.56	0.008	2.18	0.008

**Table 8:** Significance table; statistical testing (*t*-test) between the means.

Item	PM2.5	PM10	NO_2_	O_3_	SO_2_	CO
*p*-value	0.806	0.621	2.4 × 10^−12^	1.82 × 10^−5^	0.971	2.2 × 10^−16^
95% ci interval	−0.863, 0.677	−0.634, 2.680	−5.141, −5.063	0.01, 0.003	−0.102, 0.106	−0.103, −0.072
Mean difference	−0.095	0.523	−4.102	0.002	0.001	−0.087
